# Extracellular vesicles affecting embryo development *in vitro*: a potential culture medium supplement

**DOI:** 10.3389/fphar.2024.1366992

**Published:** 2024-09-18

**Authors:** Yamei Xue, Haixia Zheng, Yuping Xiong, Kun Li

**Affiliations:** ^1^ Assisted Reproduction Unit, Department of Obstetrics and Gynecology, Sir Run Run Shaw Hospital, School of Medicine, Zhejiang University, Hangzhou, China; ^2^ Institute for Reproductive Health, School of Pharmacy, Hangzhou Medical College, Hangzhou, China

**Keywords:** extracellular vesicles, exosomes, cellular communication, culture media, embryo development, mechanism

## Abstract

Extracellular vesicles (EVs) are nanometer-sized lipid bilayer vesicles released by cells, playing a crucial role in mediating cellular communication. This review evaluates the effect of EVs on early embryonic development *in vitro* by systematically searching the literature across three databases, Embase, PubMed, and Scopus, from inception (Embase, 1947; PubMed, 1996; and Scopus, 2004) to 30 June 2024. A total of 28 studies were considered relevant and included in this review. The EVs included in these investigations have been recovered from a range of sources, including oviduct fluid, follicular fluid, uterine fluid, seminal plasma, embryos, oviduct epithelial cells, endometrial epithelial cells, amniotic cells, and endometrial-derived mesenchymal stem cells collected primarily from mice, rabbits, cattle and pigs. This diversity in EV sources highlights the broad interest and potential applications of EVs in embryo culture systems. These studies have demonstrated that supplementation with EVs derived from physiologically normal biofluids and cells to the embryo culture medium system has positive effects on embryonic development. Conversely, EVs derived from cells under pathological conditions have shown a negative impact. This finding underscores the importance of the source and condition of EVs used in culture media. Further, the addition of EVs as a culture medium supplement holds significant therapeutic potential for optimizing *in vitro* embryo culture systems. In conclusion, this evaluation offers a thorough assessment of the available data on the role of EVs in embryo culture media and highlights the potential and challenges of using EVs in *vitro* embryo production.

## 1 Introduction

Assisted reproductive technology (ART) has provided hope to thousands of infertile couples over recent decades. A critical step in assisted reproduction involves culturing the preimplantation embryo *in vitro*. During this process, from the fertilization of mature oocytes to the blastocyst stage, three critical events occur, including the transition from maternal to zygotic transcripts, compaction, and the first distinct lineage differentiation into inner cell mass (ICM) and trophectoderm (TE) (Hamatani et al., 2006). Embryo development *in vitro* depends on various factors, including embryo density, the concentration of embryo-secreted factors linked with the culture volume, oviductal or endometrial cells provided by co-culture systems, the stiffness of the substrate in contact with the embryo; and fluid mechanical stimulation, such as vibration and tilting, mimicked by the dynamic culture system (Gualtieri et al., 2024). Furthermore, the composition of the culture medium plays a crucial role in embryonic development, directly affecting these key developmental events and the implantation potential of embryos (Morbeck et al., 2017; Koscinski et al., 2018; Menezo and Elder, 2020). Current research and industry trends are towards using chemically defined media due to their suitability for manufacturing and monitoring. Convincing evidence has suggested that the current culture medium used in *vitro* embryo culture is suboptimal and needs improvement (Stone et al., 2014; Chronopoulou and Harper, 2015; Ducreux et al., 2023).

Recent studies have identified extracellular vesicles (EVs) as key factors influencing embryo development (Machtinger et al., 2016; Bastos et al., 2022; Chen et al., 2022; Gualtieri et al., 2024). EVs are a heterogeneous population of membranous nano to micro-sized particles secreted by almost all cell types. They contain a range of cargo, including proteins, lipids, nucleic acids, and metabolites, which can be taken up by other cells, and elicit a broad variety of biological effects (Alminana et al., 2017; Alminana and Bauersachs, 2020; Aguilera et al., 2024; Zhang et al., 2024). EVs play a crucial role in mediating communication between the female reproductive tract and gametes/embryos. For example, EVs in the maternal oviduct communicate with both male and female gametes, whereas those in the uterus communicate with male gametes and embryos. The ciliated and secretory cells of the oviduct epithelial lining that can produce the EVs, along with the oviduct fluid, establish the culture system for early embryonic development *in vivo* (Coy et al., 2012; Alminana and Bauersachs, 2020). This interaction, facilitated by endocrine and paracrine signaling factors, provides an optimal environment for the normal development of early embryos (Ezzati et al., 2014). It is further hypothesized that EVs may be beneficial for the adequate developmental competence of embryos *in vitro* culture (Machtinger et al., 2016; Gualtieri et al., 2024).

Therefore, EV supplementation in culture media may offer a viable option to optimize the embryo culture medium system and enhance IVF efficiency. This review aims to discuss the influence of EVs from various origins on embryo development and to summarize the currently available evidence on the effect of EVs as a potential supplementing component of culture medium on embryo development *in vitro*.

## 2 Search methods

To identify relevant studies, we performed a systematic search across three major databases: Embase (Elsevier, Amsterdam, Netherlands), PubMed (NCBI, Bethesda, MD), and Scopus (Elsevier, Amsterdam, Netherlands). This search was restricted to studies published in English from the inception of Embase (1947), PubMed (1996), and Scopus (2004) to 30 June 2024. We used a combination of search terms (“extracellular vesicles” OR “microvesicles” OR “microparticles” OR “exosomes” OR “epididymosomes” OR “prostasomes” OR “oviductosomes” OR “uterosomes”) AND (“embryo*” OR “development”) AND (“add*” OR “supplement*”). The detailed search approach is supplied in Supplementary Table S1.

**FIGURE 1:**
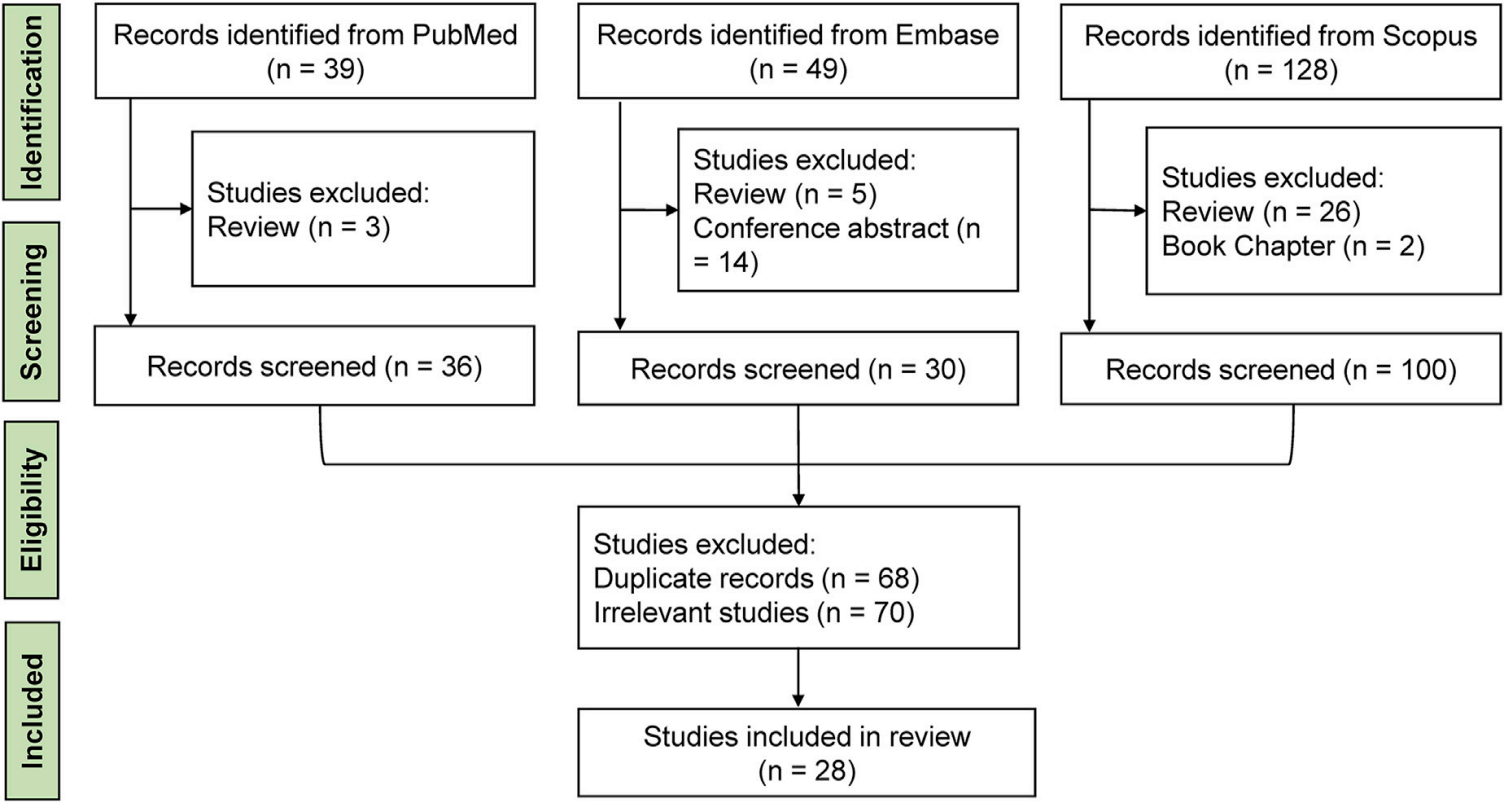
Workflow diagram of the literature review procedure.

Initially, two reviewers (YMX and HXZ) independently screened publications based on titles and abstracts, excluding irrelevant and duplicate studies. Any discrepancies were resolved through discussion with a third reviewer (KL). Full-length articles that focused on the supplementation of culture medium with EVs and their impact on embryo development *in vitro* were retrieved for further evaluation. Studies examining the effects of EVs on sperm function, egg development, or specific components like microRNAs were excluded. We also excluded meta-analyses, letters, reviews, conference proceedings, and theses. Selected articles’ references were examined to find further relevant studies. Ultimately, 28 records were deemed relevant and included in this review. The workflow of the literature review is illustrated in Figure 1. From each selected study, we extracted data on the year of publication, authors, species, origins of EVs, centrifugal force, EVs diameter range, EVs markers, the added concentration of EVs, embryo stage, embryo assessment, and main findings.

## 3 Effects of EVs derived from different origins on embryonic development *in vitro*

The effects of EVs from different origins on embryonic development *in vitro* are summarized in Table 1, with their characteristics detailed in Table 2.

**TABLE 1:** Effect of extracellular vesicle supplement on embryonic development *in vitro*.

Species	Origins of EVs	Added concentration of EVs	Starting point of co-culture embryos with EVs	Embryo assessment	Main findings	References
Mice	Endometrial-derived mesenchymal stem cells (endMSCs)	10, 20, 40 and 80 μg/mL	2-cell embryos	The percentage of expanded and hatching blastocyst, total cell number	The addition of EVs derived from endMSCs to the embryo medium significantly increased the blastocyst’s total cell number and enhanced embryo developmental competence and embryo hatching	Blazquez et al. (2018)
Bovine	Endometrial and amniotic cells	100 × 10^6^ EV/mL	Zygote, cleavage-stage embryos, and blastocyst	Blastocyst formation rate, blastomere viability, ratio of ICM/TE, gene expression analysis	The addition of microvesicles derived from amniotic cells resulted in a higher blastocyst production rate and a higher number of ICM cells and changed the expression of genes related to embryo quality	Perrini et al. (2018)
Mice	Endometrial-derived mesenchymal stem cells (endMSCs)	10, 20, 40 and 80 μg/mL	Zygotes	Total cell number, gene expression analysis	The supplementation of culture medium with EVs from endometrial-derived mesenchymal stem cells increased the quality of aged murine embryos	Marinaro et al. (2019)
Bovine	Amniotic cells	100 × 10^6^ EV/mL	Blastocyst	Hatching percentage of blastocysts, pregnancy rate	The addition of amniotic microvesicles improved the hatching and pregnancy percentages of *in vitro* bovine embryos and regulated microRNA expression	Lange-Consiglio et al. (2020)
Bovine	Uterine fluid (UF) and culture medium of endometrial epithelial cells (EVC)	4.8 × 10^8^ EV/mL	Morulae	The rates of expanded and hatched, the diameter of a blastocyst, and gene expression analysis	Embryos treated by the EV-UF tended to have a higher percentage of expanded blastocysts on day seven and hatched blastocysts on day nine compared to the control. EV-UF treatment induced a sustained increase in diameter during embryonic development	Aguilera et al. (2024)
Porcine	Oviductal fluid (OF)	7.3 × 10^8^, 7.3 × 10^9^, 7.3 × 10^10^ EV/mL	Parthenogenetically activated embryos	Cleavage rate, blastocyst yield, cell number in blastocyst, apoptosis	Oviductal EVs supplementation significantly increased blastocyst formation rate and total cell numbers, attenuated apoptosis, and relieved endoplasmic reticulum stress in the parthenogenetically activated embryos	Fu et al. (2022)
Porcine	Oviductal fluid (OF)	0.2 mg protein/mL	Zygotes	Cleavage, blastocyst and hatching rates, the number of cells per blastocyst	EVs from oviductal and uterine fluids supplementation in sequential *in vitro* culture significantly improved blastocyst total cell number, survival rate after vitrification, lipid content, and relative changes in expression of lipid metabolism transcripts and lipase activation	de Alcantara-Neto et al. (2022)
Bovine	Oviductal fluid (OF)	3 × 10^5^ EV/mL	Zygotes	Cleavage rate, blastocyst yield, survival rate	Blastocysts cultured with isthmus EVs by ultracentrifugation at 100,000 g had a significantly higher survival rate after vitrification than the other groups	Lopera-Vasquez et al. (2017)
Bovine	Oviductal fluid (OF)	0.05 mg of proteins/mL	Zygotes	Cleavage rate; blastocyst yield, blastocyst hatching, cell numbers per blastocyst, embryo phospholipid profiles	Oviductal EVs did not affect the rates of cleavage and blastocyst yield, cell numbers per blastocyst, or the rates of blastocyst re-expanding and hatching after thawing but changed the phospholipid composition of bovine embryos developed *in vitro*	Banliat et al. (2020)
Bovine	Oviductal fluid (OF) and Uterine fluid (UF)	3 × 10^5^ EV/mL	Zygotes	Number of cells per blastocyst, mitochondrial activity, lipid content, relative mRNA abundance, protein level, lipid quantification in blastocyst	Loss of EV renewal in the *in vitro* culture (IVC) system negatively affected EVs’ role in embryonic autophagy, epigenetics, and apoptosis, while the gain of EV renewal in the IVC system reduced the adverse effects and ensured the beneficial function of EVs	Leal et al. (2022)
Bovine	Ampullary oviduct fluid (AOF) and Follicular fluid (FF)	NA	Fertilized oocytes	Cleavage rate; blastocyst yield, embryo cell number (ICM, TE, and ICM/TE ratio), and apoptotic cell ratio	Supplementation with EVs isolated from FF and AOF by density gradient ultracentrifugation significantly increased blastocyst yield and total cell number and reduced apoptotic cell ratio	Asaadi et al. (2021)
Rabbit	Oviduct fluid	1.87 × 10^10^; 1.87 × 10^11^, and 1.87 × 10^12^ EV/mL	Fertilized oocytes	Blastocyst rate, Levels of ROS and 5-mC, apoptotic indexes, ICM/TE ratios, and gene expression	The addition of either EVs (1.87 × 10^11^ particles/mL) or melatonin (340 ng/mL) led to a significant downregulation of ROS and 5-mC, as well as an increase in the blastocyst rate of embryos. A combination of EVs (1.87 × 10^10^ particles/mL) and melatonin (34.3 pg/mL) resulted in the same results as well as a significant decrease in the apoptosis index and an increase in ICM/TE ratio	Qu et al. (2020)
Rabbit	Oviduct fluid	9.1 × 10^10^, 9.1 × 10^11^, 9.1 × 10^12^ EV/mL	Zygotes	ROS level, mRNA expression, H3K36me3 intensity, LC3 intensity, apoptosis index, and total cell number per blastocyst	EVs from OEC supplementation significantly increased blastocyst formation and hatching rates and decreased ROS levels, apoptosis, and blastocyst lipid content	Qu et al. (2022)
Porcine	Oviduct epithelial cell (OEC)	50 μg protein/mL	Parthenogenetic and cloned embryos	Cleavage rate; blastocyst yield, blastocyst hatching, apoptosis	Oviductal EVs supplementation in embryo culture media improved blastocyst development, total cell number, and rate of embryo hatching	Fang et al. (2022b)
Porcine	Oviduct epithelial cell (OEC)	50 ng protein/mL	Parthenogenetically activated embryos	Cleavage rate; blastocyst yield, blastocyst hatching, blastocyst cell number, blastocyst attachment, gene expression	Treatment with EVs from OEC significantly increased blastocyst formation and hatching rates, ICM/TE ratio, and attachment ability of embryos	Fang et al. (2022a)
Porcine	Oviduct epithelial cell (OEC)	1.5 × 10^7^ EV/mL	Parthenogenetically activated embryos	Cleavage rate; blastocyst yield, blastocyst cell number, blastocyst apoptosis	EVs derived from steroids-primed OEC significantly improved blastocyst formation, ICM/TE ratio, total cell number, and reduced blastocyst apoptosis	Bang et al. (2023)
Porcine	Oviduct epithelial cell (OEC)	50 ng protein/mL	Fertilized oocytes	Cleavage rate; blastocyst yield, blastocyst cell number, cortical granule distribution, embryo mitochondrial activity	EVs from OEC significantly enhanced oocyte mitochondrial activity, decreased the polyspermy rate, and increased cleavage and blastocyst formation rates of IVF embryos	Fang et al. (2023)
Bovine	Oviduct epithelial cell (OEC)	3 × 10^5^, 1.5 × 10^5^, 0.75 × 10^5^ EV/mL	zygotes	Cleavage rate, blastocyst yield, survival rate, embryo cell number	Embryos cultured in the presence of EVs had more trophectoderm and total cells and a higher survival rate after vitrification than a control group	Lopera-Vasquez et al. (2016)
Bovine	Bovine oviduct epithelial cell (BOEC)	3 × 10^6^, 6 × 10^6^, 9 × 10^6^, 1.5 × 10^7^, 3 × 10^7^ EV/mL	zygotes	Cleavage rate, blastocyst yield, hatched blastocyst, reactive oxygen species (ROS) level, DNA damage level, lipid content, mitochondrial function	The addition of BOECs-conditioned media and its isolated exosomes significantly enhanced the quality and development of embryos in terms of blastocyst formation rate and hatching ability, reduced cellular stress, and improved embryo metabolism and mitochondrial functioning	Sidrat et al. (2020)
Mice	Human endometrial epithelial cell	6, 30 mg protein/mL	2-cell embryos	Blastocyst and hatching rates, embryo cell number, implantation rate	Exosomes from hormone-treated endometrial epithelial cells significantly increased blastocyst rate, total cell number, hatching rate, and implantation rate	Gurung et al. (2020)
Porcine	Embryo	NA	Cloned embryos	Cleavage rate, blastocyst formation, blastocyst cell count	Co-culturing of cloned embryos with parthenogenetic embryos significantly increased the cleavage and blastocyst formation rates of cloned embryos. Exosomes/microvesicles derived from parthenogenetic embryos may mediate and improve the embryos’’ development	Saadeldin et al. (2014)
Bovine	Embryo	NA	Cloned embryos	Blastocyst formation rate, total cell number, ratio of ICM/TE	The supplementation of embryo-derived exosomes is essential for blastocyst formation and the quality of cloned embryos. Exosome removal impairs the development of cloned embryos	Qu et al. (2017)
Bovine	Follicular fluid	NA	Zygotes	Blastocyst rate, DNA methylation, and hydroxymethylation	Supplementation of EVs from FF of 3–6 mm follicles caused a significant increase in blastocyst rate and change in global DNA methylation and hydroxymethylation compared with the control group	da Silveira et al. (2017)
Bovine	Uterine fluid	NA	Cloned embryos	Blastocyst and hatching rates, inner cell mass/trophectoderm cell ratio, apoptosis index	Uterus-derived exosome supplementation significantly increased blastocyst and hatching rates, ICM/TE ratio, and decreased apoptosis index	Qiao et al. (2018)
Mice	Endometrial from patients with recurrent implantation failure (RIF)	5, 10, and 20 μg/mL	2-cell embryos	Blastocyst rate, hatching rate, and invasion rate	Endometrial EVs from patients with RIF attenuate embryonic development by reducing the blastocyst rate, hatching rate, and total cell number of the blastocyst, inhibiting the invasion capacity of hatched embryos	Liu et al. (2020)
Mice	Seminal plasma	NA	Zygotes	Fertilization rate, cleavage rate, blastocyst rate, apoptosis, and ICM/TE ratios	The addition of seminal plasma EVs significantly increased the rate of blastocyst formation and ICM/TE ratio and reduced the apoptosis of blastocysts	Ma et al. (2022)
Mice	Human fallopian tubal fluid	1 × 10^9^, 1 × 10^10^, 1 × 10^11^ EV/mL	2-cell embryos	Blastocyst and hatching rates, total cell numbers, inner cell mass cell proportions, relative expression levels of ROS, apoptotic cell proportions, and gene expression	The addition of human oviductal EVs Co-culturing embryos with EVs derived from human fallopian tubal fluid significantly increased the blastocyst rate, hatching rate, as well as total cell number of blastocysts and significantly decreased the levels of ROS and apoptotic cell proportions	Li et al. (2023b)
Mice	Human Fallopian tubal fluid from women with endometriosis	1 × 10^10^ EV/mL	Zygote	Blastocyst rate, relative gene expression, relative expression level of ROS, MMP, total cell number, and apoptotic cell proportion	Oviductal EVs from patients with endometriosis negatively influence early embryo development by down-regulating oxidative phosphorylation	Li et al. (2023a)

NA, not available; OEC, oviduct epithelial cell; OF, oviductal fluid; RIF, recurrent implantation failure; BOEC, bovine oviduct epithelial cell; IVC, *in vitro* culture; ICM/TE: Inner cell mass/trophectoderm; AOF, ampullary oviduct fluid; OEC, oviduct epithelial cell; UF, uterine fluid; GC, granulosa cell; SCNT, somatic cell nuclear transfer; FF, follicular fluid. ROS, reactive oxygen species; 5-Mc, 5-methylcytosine; endMSCs, endometrial-derived mesenchymal stem cells.

**TABLE 2:** Characterization of extracellular vesicles from different origins.

Origins	Species	Centrifugal force	Diameter	Markers	Concentration of EVs (Particles/mL)	References
Oviduct fluid	Rabbit	100, 000 g for 70 min	NA	NA	1.87 × 10^10^ ± 1.37 × 10^9^	Qu et al. (2020)
	Rabbit	100, 000 g for 2 h	30–500 nm	CD63, CD9	9.1 × 10^10^	Qu et al. (2022)
	Porcine	NA	43–411 nm	CD63, CD9	NA	Fu et al. (2022)
	Porcine	100, 000 g for 90 min	Small EVs: 30–100 nm; large EVs: 100–1,000 nm	TSG101, FLOT1, and Annexins 1, 4, and 5	NA	de Alcantara-Neto et al. (2022)
	Bovine	100, 000 g for 90 min	NA	NA	NA	Banliat et al. (2020)
	Bovine	10, 000 g for 60 min; 100, 000 g for 60 min	<200 nm (100, 000 g); >500 nm (10, 000 g)	ERM, TSG101, and CD9	Ampulla: (9.0 ± 3.4) × 10^8^ (10, 000 g) and (10.5 ± 3.1) × 10^8 (^100, 000 g) Isthmus: (3.6 ± 2.5) ×10^8^ (10, 000 g) and (7.5 ± 0.2) ×10^8^ (100, 000 g)	Lopera-Vasquez et al. (2017)
	Human	120, 000 g for 90 min	Mean size: 144.4 nm (control) and 148.4 nm (experiment)	CD9 and TGS 101	3 × 10^11^–1 × 10^12^	Li et al. (2023a)
	Human	120,000 g for 90min	124.5–152.6 nm	ALIX, TGS101, CD9	1.2×10^10^–3.80×10^11^	Li et al. (2023b)
Oviduct epithelial cell (OEC)	Bovine	100, 000 g for 60 min	150–200 nm	CD9, CD63, TSG101, and ERM	3 × 10^5^	Lopera-Vasquez et al. (2016)
	Bovine	100, 000 g for 60 min	80–150 nm	CD9	3 × 10^8^	Sidrat et al. (2020), Wei et al. (2022)
	Porcine	100, 000 g for 60 min	122.2 ± 1.0 nm	CD9, CD63, CD81, and flotillin	(2.46 ± 3.14) × 10^8^	Fang et al. (2022a)
	Porcine	100, 000 g for 60 min	151.9 ± 1.1 nm	NA	2.05 × 10^10^ ± 4.78 × 10^7^	Fang et al. (2022b)
	Porcine	100, 000 g for 180 min	223.8 ± 6.1 nm, 208.0 ± 4.4 nm, 242.2 ± 4.3 nm, 181.5 ± 4.7 nm, 226.2 ± 2.8 nm and 188.5 ± 0.3 nm for different groups	CD9, CD63, CD81	NA	Bang et al. (2023)
	Porcine	NA	146.2 ± 57.2 nm	NA	5.3 × 10^9^	Fang et al. (2023)
Embryo	Porcine	200, 000 g for 2 h	Early embryonic stage: 35.4 ± 6.9 nm; late embryo stage: 101.66 ± 18.4 nm	CD9	NA	Saadeldin et al. (2014)
	Bovine	100, 000 g for 70 min	60–150 nm	CD9	NA	Qu et al. (2017)
Endometrial epithelial cell	Human	120, 000 g for 60 min	100 nm	Alix, TSG101, and CD9	NA	Liu et al. (2020)
	Human	100, 000 g for 1 h	NA	Alix, TSG101	NA	Gurung et al. (2020)
Endometrial-derived mesenchymal stem cells (endMSCs)	human	4,000 g for 1 h	153.5 ± 63.05 nm	CD9 and CD63	3.31 × 10^11^ ± 3.8 × 10^9^	Blazquez et al. (2018)
	Human	4,000 g for 1 h	153.5 ± 63.05 nm	CD9 and CD63	3.31 × 10^11^ ± 3.8 × 10^9^	Marinaro et al. (2019)
Follicular fluid (FF)	Bovine	100, 000 g for 70 min	30–200 nm	ALIX, CD63	NA	da Silveira et al. (2017)
Seminal plasma	Mice	100, 000 g for 70 min	100–200 nm	NA	6.07 × 10^10^ ± 2.91 × 10^9^	Ma et al. (2022)
Follicular (FF) and ampullary oviduct fluid (AOF)	Bovine	100, 000 g for 3 h	40–400 nm	CD63, CD9, and TSG101	(2.4 ± 0.2) × 10^12^ - (1.8 ± 0.2) × 10^13^	Asaadi et al. (2021)
Oviductal (OF) and uterine fluid (UF)	Bovine	100, 000 g for 30 min	Mean size: 177.5 nm (OF) and 216.5 nm (UF)	CD9, HSP70, and ALIX	2.97 × 10^10^ (OF), 7.98 × 10^10^ (UF)	Leal et al. (2022)
Uterine fluid (UF)	Bovine	100, 000 g for 2 h	50–150 nm	CD9	NA	Qiao et al. (2018)
Endometrial and amniotic cells	Bovine	100 000 g for 1 h	258 ± 55 nm (amniotic cells), 238 ± 40 nm (endometrial cells)	NA	540 × 10^6^ (amniotic cells), 670 × 10^6^ (endometrial cells)	Perrini et al. (2018)
Amniotic cells	Bovine	100,000 g for 1 h	275 ± 8.4 nm	NA	NA	Lange-Consiglio et al. (2020)
Uterine fluid (UF) and culture medium of endometrial epithelial cells (EVC)	Bovine	100,000–120,000 g for 120 min	154.9 ± 20.8 nm (UF) and 236.7 ± 22.5 nm (EVC)	CD9, CD81, CD63 and CD40	1.20e + 010 ± 2.94e + 009 (UF) and 1.32e + 010 ± 3.02e + 009 (EVC)	Aguilera et al. (2024)

NA, not available; CD63: CD63 molecule; CD9, CD9 molecule; CD81, CD81 molecule; TGS, 101, tumor susceptibility gene 101; FLOT1, human flotillin 1; HSP70, heat-shock protein 70; ALIX, apoptosis-linked gene two-interacting protein X; OF, oviductal fluid; GC, granulosa cell; UF, uterine fluid; FF, follicular fluid; endMSCs, endometrial-derived mesenchymal stem cells.

### 3.1 EVs derived from oviductal fluid

Oviductal fluid containing EVs, known as oviductosomes (Al-Dossary et al., 2013), is the first environment encountered by a mammalian embryo. The oviduct, comprising the infundibulum, ampulla, and isthmus, is where fertilization and early embryo development occur. Numerous studies have evaluated the effects of oviductal fluid EVs on the development and quality of *in vitro*-cultured embryos (Lopera-Vasquez et al., 2017; Banliat et al., 2020; Qu et al., 2020; Asaadi et al., 2021; de Alcantara-Neto et al., 2022; Fu et al., 2022; Leal et al., 2022; Qu et al., 2022; Li et al., 2023a; Li et al., 2023b). Research has spanned to various mammals, including humans (Li et al., 2023a; Li et al., 2023b), rabbits (Qu et al., 2020; Qu et al., 2022), pigs (de Alcantara-Neto et al., 2022; Fu et al., 2022), and cattle (Lopera-Vasquez et al., 2017; Banliat et al., 2020; Asaadi et al., 2021; Leal et al., 2022). For instance, Lopera-Vásquez et al. demonstrated that adding bovine oviduct EVs to the *in vitro* culture of bovine embryos improved blastocyst quality compared to serum-supplemented media (Lopera-Vasquez et al., 2017). These EVs also upregulated genes related to metabolism, epigenetics, and water channels (Lopera-Vasquez et al., 2017). Asaadi and colleagues found that oviduct fluid EVs derived by OptiPrep™ density gradient ultracentrifugation (ODG UC) positively impacted embryo quality compared to EVs isolated by single-step size exclusion chromatography (Asaadi et al., 2021). Leal et al. showed that EV supplementation in a sequential culture system improved cell number, reduced lipid content, and increased post-vitrification survival of bovine embryos (Leal et al., 2022). Banliat et al. discovered that oviductal EVs induced significant changes in the phospholipid composition of embryos, suggesting a role in embryonic lipid metabolism (Banliat et al., 2020). Qu et al. found that melatonin in oviductal fluid and EVs increased the blastocyst rate and reduced ROS and 5-methylcytosine levels. The authors also demonstrated that the beneficial effect of oviductal fluid and its EVs was inhibited by luzindole, a melatonin receptor agonist (Qu et al., 2020). Fu et al. confirmed that oviductal EVs alleviate endoplasmic reticulum stress, benefiting early embryonic development (Fu et al., 2022). However, EVs added to the culture medium may degrade, leading to decreased embryonic development competence and increased oxidative stress, autophagy imbalance, and abnormal epigenetic modification (Qu et al., 2022). Renewing EVs in the culture system significantly reduced these adverse effects (Qu et al., 2022). De Alcântara-Neto et al. found that 2 days of EV treatment enhanced cleavage and blastocyst rates (de Alcantara-Neto et al., 2022). These EVs, released from oviductal epithelial cells and blood transudation, are internalized by embryos and influence their development. Co-culturing murine embryos with EVs derived from human fallopian tubal fluid significantly increased the blastocyst rate, hatching rate, and total cell number of blastocysts while decreasing the ROS levels and apoptotic cell proportions (Li et al., 2023b). However, oviductal EVs from patients with endometriosis negatively influence early murine embryo development by down-regulating oxidative phosphorylation (Li et al., 2023a).

### 3.2 EVs derived from oviduct epithelial cells (OEC) cultured *in vitro*

The oviduct epithelium, consisting of ciliated and secretory cells, produces the oviduct fluid that supports fertilization and early embryonic development. EVs secreted by OEC cultured *in vitro* have been isolated and characterized through various techniques, including transmission electron microscopy and proteomics. Adding EVs from OECs to *in vitro* culture media positively affected embryo development in mammals such as pigs and cattle (Lopera-Vasquez et al., 2016; Sidrat et al., 2020; Fang et al., 2022a; Fang et al., 2022b; Wei et al., 2022; Bang et al., 2023; Fang et al., 2023). Lopera-Vásquez et al. first reported that EVs from bovine OECs improved the quality of embryos cultured *in vitro*, suggesting that EVs mediated oviduct-embryo communication (Lopera-Vasquez et al., 2016). Sidrat et al. revealed that exosomes from bovine oviduct epithelial cells (BOEC) re-establish pyruvate flux, improving mitochondrial function during preimplantation development (Sidrat et al., 2020). Wei et al. found that exosomes from BOECs significantly improved oocyte maturation, early embryo development, and implantation potential (Wei et al., 2022). Fang et al. demonstrated that EVs from OEC increased the rate of embryo breakage and the ICM/TE ratio of porcine embryos, enhancing reprogramming and implantation-related genes (Fang et al., 2022a). Another study by Fang et al. showed that OEC EVs improved the quality and developmental ability of porcine parthenogenetic and cloned embryos (Fang et al., 2022b). Additionally, Fang et al. demonstrated that EVs isolated from OEC improved the cortical granules concentration and mitochondrial activity of porcine oocytes (Fang et al., 2023). Furthermore, the secretion of EVs may be associated with the hormonal environment in cell culture. Bang and colleagues mimicked *in vivo* hormonal conditions and treated porcine OECs with estradiol and progesterone, finding that EVs from conditioned OECs decreased apoptotic rate that, in turn, contributed to improved blastocyst development (Bang et al., 2023). EV characteristics of OEC linked with the hormonal condition are confirmed by our previous study, showing that the size and cargoes of EVs from mouse oviduct fluids are influenced by the estrous cycle (Yi et al., 2022). These results imply that hormones may regulate the effect of EVs from OEC on embryo development.

### 3.3 EVs derived from endometrial epithelial cells cultured *in vitro*

The complex process of embryo implantation calls for effective communication between the endometrium and the embryo (Kurian and Modi, 2019). EVs have been identified as crucial mediators in early pregnancy, particularly during implantation. Co-culture systems using embryos and EVs derived from endometrial epithelial cells have been developed to explore this interaction (Gurung et al., 2020). Gurung et al. investigated the role of different components of the secretome, including total secretome (TS), soluble secretome (SS), and crude exosomes from hormonally primed human endometrial epithelial cell culture medium, in mediating mouse embryo development and implantation (Gurung et al., 2020). They found that exosomes significantly enhanced the total cell number of *in vitro* embryos and the blastocyst hatching rate, suggesting that endometrial exosomes can enter embryonic cytoplasm and regulate embryo growth and implantation (Gurung et al., 2020). Liu and colleagues further explored this by isolating endometrial EVs from patients with recurrent implantation failure and fertile women, using a co-culture model to compare their effects on murine embryos (Liu et al., 2020). They found that endometrial EVs from patients with recurrent implantation failure attenuate embryonic development and decreased invasion capacity, highlighting the potentially harmful role of EVs in pathological conditions (Liu et al., 2020). Authors speculated that the molecules upregulated in the endometrial of patients with recurrent implantation failure, such as miR-145 (Revel et al., 2011; Wen et al., 2018), may transmitted to embryonic cells via EVs, thereby impeding the migrating and invasion capacity of trophoblasts (Liu et al., 2020). These findings underscore the potential of EVs from endometrial epithelial cells in improving *in vitro* culture conditions and emphasize the importance of adequate endometrium-embryo communication for successful implantation.

### 3.4 EVs from Co-Cultured embryos

Preimplantation embryo culture in groups can produce a supportive microenvironment through the secretion of various autocrine and paracrine factors, significantly enhancing embryo development (Saadeldin et al., 2014; Qu et al., 2017; Wydooghe et al., 2017). These factors are secured through active secretion, passive flow, or cargo loading into EVs (Wydooghe et al., 2017). Saadeldin et al. found that co-culturing porcine embryos derived from somatic cell nuclear transfer (SCNT) with parthenogenetic embryos improved cleavage rates and blastocyst formation, mediated by exosomes and microvesicles secreted by parthenogenetic embryos (Saadeldin et al., 2014). Similarly, Qu et al. demonstrated that supplementing the culture medium with exosomes derived from bovine SCNT embryos benefits their subsequent development, while the removal of exosomes during medium replacement impairs the development potential of SCNT embryos (Qu et al., 2017). These studies highlight the importance of EVs in creating a conducive environment for embryo development.

### 3.5 EVs from amniotic cells

Amniotic cells have been shown to create a more suitable microenvironment for embryo growth, enhancing *in vitro* embryo development. Studies have found that co-culturing embryos with amniotic cells significantly increases the blastocyst formation rate compared to co-culture with bone marrow-derived cells and cumulus cells (Lange-Consiglio et al., 2012). Perrini et al. reported that the addition of microvesicles derived from amniotic cells resulted in a higher blastocyst production rate and a higher number of ICM cells (Perrini et al., 2018). Additionally, the expression of the proapoptotic Bax gene was downregulated (Melka et al., 2010), while the expression of antioxidant selenoprotein GPX1 was significantly upregulated in the group cultured with amniotic cells (Perrini et al., 2018). Further research by the same team indicated that amniotic microvesicles improved the hatching and pregnancy percentages of *in vitro* bovine embryos and modulated the expression of specific microRNAs associated with embryo implantation (Lange-Consiglio et al., 2020). These findings suggest that amniotic cells and their derived EVs play a vital role in supporting embryo development and implantation.

### 3.6 EVs from uterine fluid

EVs released by the endometrial epithelial cells into the uterine cavity are essential for embryo development and implantation. Qiao et al. reported that exosomes from bovine uterine fluid significantly improved blastocyst yield, hatching rate, and inner cell mass/trophectoderm cell ratio while decreasing apoptosis in cloned embryos (Qiao et al., 2018). Leal et al. established a sequential *in vitro* culture system using EVs from the bovine oviduct and uterine fluids, which improved embryo quality post-thaw survival rate, total cell number and reduced lipid content (Leal et al., 2022). Aguilera et al. demonstrated that culturing embryos in a medium supplemented with EVs from uterine fluid tended to have a higher percentage of expanded blastocysts on day seven and hatched blastocysts on day nine compared to the control, with a sustained increase in diameter during embryo development (Aguilera et al., 2024). The expression of the IFNT gene related to embryo implantation was highly expressed in the group treated with EVs from uterine fluid (Aguilera et al., 2024). Collectively, the data obtained from these studies suggest that uterine fluid EVs influence embryo development and implantation potential.

### 3.7 EVs from follicular fluid

Follicular fluid EVs play a crucial role in oocyte maturation and embryo development. These EVs have been detected in various species, including equine, cats, cattle, and humans (da Silveira et al., 2012; Sohel et al., 2013; de Almeida Monteiro Melo Ferraz et al., 2020; Neyroud et al., 2022; Soares et al., 2023; Luis-Calero et al., 2024). Studies have shown that follicular fluid EVs improve blastocyst formation rates, as well as alter transcript levels and DNA methylation patterns (da Silveira et al., 2017). These impacts are thought to be mediated by particular miRNAs within the EVs that regulate DNA-protein interactions in embryos (da Silveira et al., 2017). Likewise, Asaadi et al. found that bovine embryos cultured with follicular fluid EVs had higher blastocyst yield and quality compared to control groups (Asaadi et al., 2021). These findings indicate that follicular fluid EVs enhance preimplantation embryo development and implantation potential.

### 3.8 EVs from endometrial-derived mesenchymal stem cells (endMSCs)

The cross-talk between the endometrium and embryo is essential for supporting embryo implantation, and this intercellular communication is partially mediated by EV release (Homer et al., 2017). The endMSCs are a subgroup of stem cells in endometrial tissue that produce various paracrine factors involved in cell growth and proliferation (Zuo et al., 2018). Blázquez et al. first reported that the addition of EVs derived from endMSCs to embryo medium significantly increased the blastocyst’s total cell number and enhanced embryo developmental competence and hatching (Blazquez et al., 2018). Further research by the same team demonstrated a beneficial effect on the development of embryos from aged female mice with supplementation of EVs derived from endMSCs (Marinaro et al., 2019). Gene expression analysis in the resulting blastocysts showed significant changes in key genes involved in metabolism, placentation, the oxidative stress response in cells, and the development of trophectoderm and inner cell masses (Marinaro et al., 2019). These findings highlight the potential of endMSC-derived EVs in supporting embryo development and implantation.

### 3.9 Other origins of EVs

Seminal plasma, originating from the male accessory glands and the epididymal duct, plays a significant role in modulating the maternal environment during natural pregnancy (Druart and de Graaf, 2018; Schjenken and Robertson, 2020). EVs from seminal plasma are believed to contribute to early embryo development by mediating signal communication between seminal plasma and the embryo. Ma et al. found that adding EVs from mouse seminal plasma to the embryo culture medium decreased blastocyst apoptosis and increased blastocyst formation rates and inner cell mass/trophectoderm cell ratios, suggesting improved embryo development (Ma et al., 2022). These investigations underscore the importance of seminal plasma EVs in supporting early embryonic growth.

In summary, supplementing culture media with healthy EVs derived from various origins, including oviduct fluid, follicular fluid, uterine fluid, seminal plasma, embryo, oviduct epithelial cells, endometrial epithelial cells, amniotic cells, and endometrial-derived mesenchymal stem cells, appears to enhance the developmental capacity and implantation potential of embryos. This is evidenced by increased total cell numbers, higher blastocyst yield and hatching rates, decreased ROS levels and fewer apoptotic cells. These findings emphasize the positive influence of EVs on *in vitro* embryo development, suggesting that EVs would serve as the supplement of *in vitro* culture media.

## 4 Mechanisms of EVs functioning in embryonic development

EVs can be internalized by embryonic cells *in vitro*. They can pass through the zona pellucida and subsequently be internalized by embryonic cells, positively influencing development and significantly enhancing blastocyst formation, hatching rates, and total cell numbers (da Silveira et al., 2017; Qu et al., 2020; Asaadi et al., 2021). The process of EV-mediated intercellular signaling process involves three main steps: EV release from donor cells, EV uptake by recipient cells, and targeted delivery of cargo to recipient cells (Wan et al., 2022). The mechanisms by which cells uptake EVs depending on their source and the cell type, including clathrin-mediated endocytosis, macropinocytosis, lipid raft-mediated endocytosis, receptor-mediated endocytosis, and direct membrane fusion (Mulcahy et al., 2014; Esmaeili et al., 2022).

EVs carry a variety of cargoes. The two main types of cargo are proteins and nucleic acids, which are crucial during the peri-implantation stage (Kurian and Modi, 2019; Fu et al., 2020; Nakamura et al., 2020). Protein cargoes in EVs are associated with many biological processes related to fertilization and embryonic development. Proteins associated with fertilization include oviduct glycoprotein (OVGP1), heat-shock protein family HSP90, HSPA8, HSP70, and myosin 9 (MYH) (Kadam et al., 2006; Elliott et al., 2009; Alminana et al., 2017; Zhao and Kan, 2019; Braganca et al., 2021; Lee et al., 2021; Zhao et al., 2022). Proteins involved in embryonic development include those regulating: (1) gene expression as potential biomarkers, PAG1, IFN-T, and PLAC8; (2) energy and metabolism, PLIN2, ACACA, LDHA, LDLR, PPARGC1B, FASN, and PNPLA2; (3) epigenetic regulation, DNMT3A, H3K36me3, TFAM, SNRPN; (4) oxidative stress, GPX1, MnSOD, GLUT1, GAPDH, GPX3, SOD2, GSTM2, miR-126, miR-21, and miR-128; and (5) autophagy, ATG5, BECN1, and MAP1LC3C (Lopera-Vasquez et al., 2016; Lopera-Vasquez et al., 2017; Leal et al., 2022; Qu et al., 2022). Nucleic acid cargoes in EVs that have been studied can be divided into two types of RNA: miRNA (de Alcantara-Neto et al., 2022; Leal et al., 2022) and transfer RNA-derived small RNAs (tsRNAs) (Fan et al., 2024). Li et al. identified 79 miRNAs commonly present in all EV samples out of 617 known miRNAs, with target genes involved in several signaling pathways, including the p53, Ras, PI3K/AKT, and Hippo pathways, which are essential for embryonic cell division, differentiation, and development. Examination of the transcript levels of the genes connected to embryo development showed significant upregulation in levels of Actr3, Eomes, and Wnt3a (Li et al., 2023b). Fan et al. (2024) revealed the abundance of tsRNAs in EVs secreted by blastocysts, and inhibition of tDR-14:32-Glu-CTC-1 promotes embryo hatching while influencing embryo implantation-related genes and pathways (Fan et al., 2024).

EVs regulate diverse critical functions during embryo development through multilevel intercellular signaling. The signaling encompasses cell division, growth, metabolism, and apoptosis during early embryo development (Alminana and Bauersachs, 2020; de Alcantara-Neto et al., 2022; Qu et al., 2022). EVs regulate crucial cellular activities such as adhesion, migration, and proliferation (Ng et al., 2013; Zdravkovic et al., 2015). EVs interact with target cells to promote embryonic cell growth, division, and implantation (Bastos et al., 2022; Chen et al., 2022). They significantly promote embryo implantation by up-regulating reprogramming genes related to embryonic cell proliferation and differentiation, such as POU5F1, NANOG, SOX2, c-MYC, and Klf4 (Jaber et al., 2017; Hassani et al., 2019; Fang et al., 2022a). Comprehensive quantitative proteomic profiling has revealed changes in human trophectodermal spheroids, including adhesion proteome and function, regulated by the transfer of endometrial EV cargo proteins to the trophectoderm (Evans et al., 2019). Similar to proteins in EVs, miRNAs encapsulated in EVs also modulate target-cell function by affecting the expression of transcripts and proteins during early embryo development, influencing embryo growth, morphology, and implantation, and emerging as crucial mediators of cross-talk during peri-implantation (Ng et al., 2013; Vilella et al., 2015; Balaguer et al., 2018).

Taken together, EVs, in the unique microenvironment of early embryonic development, are internalized by embryonic cells, carrying a variety of cargoes essential for development. Once internalized, EVs regulate a range of critical functions within embryos. The process of EV-mediated intercellular signaling facilitates communication and signaling that are essential for successful growth and enhances trophectoderm adhesion and invasion, thereby increasing implantation potential.

## 5 Future directions on clinical implications: optimizing *the in vitro* culture media

The success of IVF treatments depends on the quality of embryos, especially as the number of embryos transferred in clinics decreases. Unfortunately, embryos cultured *in vitro* often exhibit lower blastocyst formation rates, reduced pregnancy rates, and higher fetal loss compared to those developed *in vivo*. Clinical observations have identified the transfer of low-quality embryos as a major cause of implantation failure in IVF treatments (Abel et al., 2019; Bori et al., 2022). Some patients even lack the opportunity for embryo transfer due to the absence of high-quality embryos. Therefore, optimizing the conditions affecting embryo development in IVF labs is essential.

The supplementation of EVs in the *in vitro* culture media may be one promising strategy for enhancing embryo development. Despite the use of various culture media (Sepulveda et al., 2009; Paternot et al., 2010; Hardarson et al., 2015), the optimal culture medium remains unclear (Utsunomiya et al., 2022). The hypothesis that the lack of communication between female reproductive tracts and gamete/embryo contributes to the suboptimal competence of embryos cultured *in vitro* has led to the exploration of EV supplementation. EVs from sources such as oviduct fluid, oviduct/endometrium epithelial cells, uterine fluid, follicular fluid, embryo, and seminal plasma have shown positive effects on embryo development when added to IVF culture systems. Conversely, EVs from pathological conditions, such as endometriosis or recurrent implantation failure, may negatively impact early embryonic development (Wang et al., 2019; Qu et al., 2022; Li et al., 2023a; Makieva et al., 2024). Thus, replacing these with normal EVs or those from different sources could improve embryonic development, making EV supplementation a prospective approach to enhance embryo quality.

EV supplementation may prepare for precise and personalized treatment in IVF. Embryonic development failure in different patients may result from pathological EVs that either lack specific cargo, such as functional proteins or have impaired functions of these cargoes. Tailoring treatments or interventions could involve using a medium supplemented with exogenous EVs that carry the necessary normal and functional cargo. These EVs can be sourced from the patient’s healthy tissues, from other healthy individuals, or through engineering strategies to customize extracellular vesicles (Esmaeili et al., 2022). Recent research has focused on the components within EVs that influence embryonic development. For instance, Benedetti et al. studied the effects of microRNA-34c from follicular fluid-derived EVs on blastocyst quality (Benedetti et al., 2024). Similarly, Cañón-Beltrán et al. investigated the impact of miR-148b from bovine oviductal EVs on early embryo development (Canon-Beltran et al., 2024). Understanding these specific components can lay the foundation for developing precise interventions using EVs with targeted molecular cargo. Additionally, studies using bovine models offer valuable insights into the potential applications of EVs in personalized medicine. These studies inform the development of tailored EV-based therapies for treating infertility in humans.

To fully harness the potential of EVs in embryo culture, standardization of EV characteristics and treatment protocols is crucial. EVs have garnered significant attention for optimizing human embryo culture media, especially for patients with no high-quality embryos across multiple treatment cycles. However, the beneficial effects of EVs depend on several factors, including their origin, concentration, size, molecular signatures, and encapsulated contents, as well as the treatment protocols of embryos, such as embryo stage, cell numbers, and treatment time. Therefore, standardized reporting guidelines for EV characteristics and embryo treatment procedures are necessary.

Despite the promise of EV supplementation, several challenges remain. The molecular mechanisms underlying the formation and release of EVs in both physiological and pathological conditions are not fully understood. It is unclear which specific molecular cargoes in EVs play a role in embryonic development and what their targets are. Most insights are derived from animal studies, and research on human embryos is ethically controversial and tightly controlled in many countries. Additionally, technological challenges limit research in this field.

## 6 Conclusion

This review summarizes the state of knowledge regarding the effects of EVs from various origins on embryo development, including oviduct fluid, oviduct/endometrium epithelial cells, uterine fluid, follicular fluid, embryos, seminal plasma, amniotic cells, and endometrial-derived mesenchymal stem cells. It provides evidence that EVs may serve as a component supplementation of *in vitro* culture media and explains the mechanism of EVs functioning in embryo development. Moreover, the future direction of EV supplementation is discussed, including potential applications and challenges. In conclusion, EV supplementation *in vitro* culture media may provide a strategy and pave the way for innovative therapies to improve human embryo development and advance clinical outcomes in ART treatment.
