# Supplementary material for: Extracellular vesicles affecting embryo development in vitro: a potential culture medium supplement
Source: Front Pharmacol. 2024 Sep 18;15:1366992. doi: 10.3389/fphar.2024.1366992 (PMC11445000; doi:10.3389/fphar.2024.1366992)
Supplement: Supplementary file 1 [file Table1.DOCX]

Table S1. Search strategy.

| Scopus  30/6/2024 | extracellular AND vesicles OR microvesicles OR microparticles OR exosomes OR epididymosomes OR prostasomes OR oviductosomes OR uterosomes AND embryo* AND development* AND add* OR supplement* | 128 |
| --- | --- | --- |
| PubMed  30/6/2024 | (((((((((((extracellular vesicles[Title/Abstract]) OR (microvesicles[Title/Abstract])) OR (microparticles[Title/Abstract])) OR (exosomes[Title/Abstract])) OR (epididymosomes[Title/Abstract])) OR (prostasomes[Title/Abstract])) OR (oviductosomes[Title/Abstract])) OR (uterosomes[Title/Abstract])) AND (embryo*[Title/Abstract])) AND (development*[Title/Abstract])) AND (add* OR supplement*[Title/Abstract]))) | 39 |
| Embase  30/6/2024 | ('extracellular vesicles':ab,ti OR microvesicles:ab,ti OR microparticles:ab,ti OR exosomes:ab,ti OR epididymosomes:ab,ti OR prostasomes:ab,ti OR oviductosomes:ab,ti OR uterosomes:ab,ti) AND embryo:ab,ti AND development:ab,ti AND (supplement:ab,ti OR addition:ab,ti OR add:ab,ti OR adding:ab,ti) | 49 |
